# Measuring the accessibility of veterinary care for companion animals in England and Wales

**DOI:** 10.1017/awf.2026.10065

**Published:** 2026-01-28

**Authors:** Stephen Clark, Graham Clarke, William James, Nik Lomax

**Affiliations:** University of Leeds, United Kingdom

**Keywords:** Accessibility, animal welfare, companion animals, competition, veterinary care, veterinary deserts

## Abstract

Recent surveys have suggested that over half of UK households own a pet. One important aspect to this ownership is ensuring that access to appropriate veterinary care is available for their pets. To measure the ease of accessibility to such care, three aspects are important, the local demand for veterinary care, the supply of care, and the ease of travel to obtain the care. For the first element, in this study estimates were made of the household pet population for all neighbourhoods in England and Wales (36,672 neighbourhoods each containing approximately 700 households). Information regarding the location and number of veterinarians working in local practices was then used, with vehicle journey times, to provide a measure of accessibility to veterinary care. It was found that the more affluent and rural locations have better accessibility to veterinary care than deprived and urban locations. The detailed geography of the estimates provided by this study enabled the location of potential ‘veterinary deserts’ to be identified. With this knowledge additional provision can be prioritised to such locations with a view to improving the welfare of companion animals. Not only will this improve the accessibility of veterinary care but, through competition, this also has the potential to reduce care costs. Thus, the likelihood of pets receiving the care they need will improve. Whilst this study focuses upon England and Wales, the methodology presented would be equally valid in other settings where appropriate data exist.

## Introduction

Many studies have pointed to the beneficial impacts of companion animal (pet) ownership on both the physical and mental health of their owners (Scoresby *et al.* 2021; Kretzler *et al.* 2022). These benefits became particularly apparent during the COVID-19 pandemic. During this time, pets enabled individuals to confidently access outdoor settings (Hoy *et al.* 2025) and provided companionship (Kretzler *et al.* 2024; von Humboldt *et al.* 2024). This period also presented challenges for animal rescue organisations, staff and volunteers, with Carroll *et al.* (2024) reporting impacts on animals (through lockdown restrictions, adoptions and relinquishments), the identity of the organisation (through inconsistency of rules and inadequate funding) and organisational processes (through increased workloads and a prioritisation of education for new pet owners).

Looking at the opportunities for emotional support and physical exercise provided by pet ownership, a recent survey report from the Competition and Markets Authority (CMA) (2025a) reported that 48% of respondents felt that their pet supported their physical health. Eighty-seven per cent of the participants also said their pet provided emotional support, with 72% saying their pet helped reduce stress or anxiety (although the mental health benefits are disputed elsewhere, e.g. Parsons *et al.* 2024). There are also studies that report how childhood pet ownership can contribute to more empathetic attitudes towards animals in later life (Paul & Serpell 1993; Miura *et al.* 2002). However, ownership of pets comes with challenges (Muldoon & Williams 2024a), some of which are direct financial challenges, such as the acquisition cost (Mead *et al.* 2024), food, and veterinary care (Muldoon & Williams 2024b). Other challenges are indirect, for example, a need to make changes to work patterns (Maris & Cameron 2023). The relationship between economic means and ownership is not clear-cut however, with one study finding that in a US context, pet ownership actually increased during times of economic recession (Crespo & Faytong-Haro 2022).

One important aspect of veterinary care is the cost. In their study of the fees posted on websites by veterinary practices for three common procedures, Egenvall *et al.* (2024) report that over the 18 months from late 2022 to early 2024, the cost of these procedures in the UK increased by between 15 and 20%. This is in the context of core UK inflation in early 2024 being around 5%. In common with many sectors of the economy, an important factor in the cost of veterinary care is associated with market competition. If households have a limited range of options regarding where to access veterinary care, then this can increase their costs. Since the COVID-19 pandemic there have been many stories in the media about high inflation in veterinary care costs (e.g. British Broadcasting Corporation 2023; Otte & Bartholomew 2023; Makortoff 2024), primarily highlighting concentration in the ownership of veterinary practices and a possible lack of alternatives in the local neighbourhood. This potential inability to access veterinary care impacts upon the provision of preventative healthcare advice and treatments for pets, leading to further pain and suffering (Croney *et al.* 2025). Left untreated, communicable diseases can lead to an increased disease burden in the general pet population.

As a result of this issue of availability of veterinary care, in September 2023 the CMA (2025b) launched a call for information from pet owners, people who work in veterinary practices and those who supply products and services to share their thoughts on the veterinary market. Following on from this in May 2024, the CMA decided to proceed with a formal market investigation into the UK’s veterinary services market for household pets. This consultation has been wide ranging and is expected to conclude in late 2025. However, some of the evidence collected has already been published by the CMA. One piece of evidence is a Working Paper reporting the findings of a survey of pet owners on available opinions surrounding veterinary care (CMA 2025a). The top two reasons mentioned for selecting a particular veterinary practice were location (68%) and recommendation from someone else (44%), and when asked to provide a main reason, 36% gave location as the main reason and 23% gave recommendations. The study reported that 55% noted there was only one choice of veterinary practice in their area and demonstrates that along with other factors, the location and choice of veterinary practices is an important component of pet health and welfare. In light of this, our study was concerned with measuring the accessibility of veterinary practices for the household pet population of England and Wales. As well as a national analysis, we focused upon a case study town (Barnsley in south Yorkshire) to illustrate the more local nuances of veterinary practice accessibility. This study did not include accessibility to care for non-owned animals (McDonald & Skillings 2021; McDonald *et al.* 2023); those owned by the homeless (Williams & Hogg 2016) or commercial livestock.

To achieve this aim, two spatial estimation techniques are used. Since there are no recent or reliable estimates of the spatial distribution of the pet population in England and Wales at the detailed neighbourhood level required here, a spatial microsimulation technique is used to estimate the number of pet-owning households in neighbourhoods roughly the size of 700 households (see *Spacial microsimulation*). The second technique is a floating catchment area (FCA) that measures a Spatial Accessibility Index (SPAI) using a combination of the pet population estimates, car journey times, the location of veterinary practices, and the number of veterinary surgeons working in each practice (see *Floating catchment area*). Profiles of accessibility by the characteristics of the neighbourhood, its deprivation and degree of urbanisation (see *Neighbourhood types*) are shown. These SPAIs are also mapped to show how accessibility varies over space, and their potential to identify ‘veterinary deserts’ is highlighted.

### Number of pet-owning households

Historic pet-owning estimates produced for 2011 by Murray *et al.* (2015) suggested that in the UK 23% of households owned one or more cats and 30% owned one or more dogs, with 7% owning both a cat and a dog. In a study conducted around the same time, Asher *et al.* (2011) estimated there to be around 9.4 million dogs in the UK, with a 95% confidence interval of 8.1 to 11.5 million.

Additionally, a number of organisations with an interest in pet health and commerce provide annual estimates of the number of pets and pet-owning households in England and Wales. These include the UK Pet Food organisation (2024) who estimate that 59% of households owned a pet in 2021, and the People Dispensary for Sick Animals (PDSA 2024) reporting that 51% of the UK population own a pet. Neither of these sources provides a spatial breakdown of pet ownership. A relatively contemporary estimate of the UK pet population that does provide estimates at a postal geography is provided by the Animal and Plant Health Agency (Aegerter *et al.* 2017). However, these were estimated in 2016 and only cover the dog and cat population. In addition, the chosen geography, postcode districts, are still large in population terms (with each, on average, containing 11,000 households in 2021). More recent estimates of just the 2019 UK dog population are available, but at an even larger postcode area geography (an average of 234,000 households) (McMillan *et al.* 2024).

The geographical distribution of pet ownership can be related to the demographic characteristics of their owners, household types and housing characteristics. For example, Murray *et al.* (2010) found that higher dog ownership levels were associated with having a garden, a larger family, and living in rural locations. For cats, higher ownership levels were associated with having a garden, having degree level qualifications and living in a semi-urban location. Other literature on household composition reports that larger households and those with older children tend to have higher levels of pet ownership (Murray *et al.* 2010; Applebaum *et al.* 2023). Purewal *et al.* (2019) were able to exploit the richness of a longitudinal survey of households to relate changes in pet ownership as families progress through certain life stages. In families, pet ownership peaked when the youngest child was 11, although for cats it remained constant until aged 18 and for dogs it increased from 11 to 18 years of age. In contrast to studies showing that families with children were more likely to have pets, a study on dog ownership reported by Anderson *et al.* (2023), showed that over three-quarters of the households owning dogs were adult-only households. A more recent study of Danish pet owners showed that socio-economic status had an influence on dog ownership, and also that living in a house and owning a property increased the tendency for dog ownership (Lund *et al.* 2024). An additional finding was that dog ownership was lower in more densely populated neighbourhoods. Other studies have also highlighted that some forms of household rental tenure can be a barrier to particular types of pet ownership, particularly in private rental properties (Simcock *et al.* 2024; Banfield-Nwachi 2025) with landlords often citing the alleged extra cleaning costs and damage to property associated with pet ownership (Propertymark 2022). There are, however, advocates of a more considered and researched approach to this issue as discussed in a recent review study by McCarthy and Simcock (2024). They find that landlords are unnecessarily resistant to allowing their tenants to have pets, and that legislative changes may bring a better balance to the expectations of both landlords and tenants.

From the literature it is apparent that there are no recent estimates of UK pet ownership at the detailed geographic scale required for accessibility calculations. The literature also suggests the most likely individual characteristics influencing pet ownership to be: life stage and economic circumstances, whilst household size, property type, tenure and neighbourhood population density also exert an influence. We shall use this information in *Pet population* to inform how we build our small area estimates of the pet population.

### Accessibility to veterinary care

Access to veterinary care has been an active area of study, especially in the US. A recent review study by Pasteur *et al.* (2024) into accessing veterinary care was, in part, looking for potential barriers to accessing such care. Of the 77 relevant international studies they identified, 35 mentioned that the geographic location was a significant challenge to accessing care for animals. At its most severe, this lack of geographic access can cultivate scenarios whereby ‘veterinary deserts’ arise in which we see underserved, or poorly served, communities. Much of the current state of the art in the notion of access to veterinary care and the concept of veterinary deserts is covered in further reviews by Bunke *et al.* (2024) and O’Connor *et al.* (2025). These studies identify at least three themes that commonly define such a desert: accessibility, which can be captured by the distance to access a practice; affordability, concerned with the cost of the care; and availability which incorporates the density and diversity of practices (these are in accordance with Penchansky and Thomas’ [1981] five dimensions of access to services in general: accessibility, affordability, availability, accommodation, and acceptability). O’Connor *et al.* (2025) additionally map various risk factors (e.g. age, social vulnerability, attitudes, and caregiver health) and the influence they may exert upon these five dimensions.

Using a nested One Health model, Lem (2019) identified three types of barriers to accessing veterinary care: socioeconomic; geographic; and knowledge-based, with an understanding that there can be interactions between these barriers. Using Geographic Information Systems (GIS) techniques, Neal and Greenberg (2022a) used two measures to identify variations in the levels of veterinary care at a US county level: one was a variety of practitioner to population ratios and a second was distance to travel calculations. These measures were also profiled by a ‘Rural Urban Commuting Areas’ classification to identify potential systematic differences in access to veterinary care. These themes were also developed into a five factor ‘Veterinary Care Accessibility Index’ by the same authors (Neal & Greenberg 2022b) with the index quantifying that there are over 21 million households in the lowest ranked quintile of accessibility, representing an estimated 25.2 million companion animals. Other recent work by Reece and Li (2023) in the city of Detroit reported an assessment of access to wider animal welfare necessities beyond just veterinary care, including pet food and supplies. Using a series of hypotheses, they found that pet resources are significantly more likely to be located in less-deprived zip codes, however the level of access was not related to having personal vehicles. Outside the US, Ng *et al.* (2022) provided a study of access to general veterinary practices and to 24/7 practices in the densely populated city of Hong Kong. They found that generally the level of access, measured as the journey time to a practice, was good. They also found some evidence for the clustering of practices providing similar services, something which is reported as a positive. In their calculations, demand was measured by the number of households in the general population, not the number of pet-owning households.

In this study we consider only the two spatial aspects to veterinary care, namely availability and accessibility. Whilst there are three common techniques to calculate accessibility in the literature, a practitioner to population ratio R (e.g. dentists per resident), a distance D to the nearest practitioner, or a count Q of the number of practitioners within a given distance or time, each has its disadvantages. The practitioner to population ratio technique assumes the existence of a defined geographic catchment area from which practitioners cannot provide services to those outside the area and those living in an area cannot access services outside the area. In the context of veterinary care in the UK this is clearly not the case, a pet owner can make use of any practice, and a practice can accept owners from any neighbourhood. The other two techniques do not take account of a competition effect for the service, it assumes that the available capacity of the provider can be used multiple times to satisfy demand. This is also not the case, all other things being equal, a neighbourhood that shared a practice with say 100 neighbourhoods is likely to provide a better service than one that shares a similar practice with 500 others. A technique that overcomes these disadvantages is Floating Catchment Areas (FCA), which is described later in *Floating catchment area.*

## Materials and methods

### Ethical status

The Faculty Research Ethics Committee (FREC) for Business, Environment, Social Sciences, University of Leeds, UK, has issued a favourable ethical opinion based on the application submitted. Research ethics application reference number 1583.

### Materials

To calculate the accessibility to veterinary practices three pieces of information are required. First, the demand population, ideally the number of pet-owning households in the neighbourhood. Second, the supply of veterinary care, both its location and capacity, and finally the ease of travel from the household to the practice.

#### Pet population

The household pet population is estimated using a combination of 2021 Census data for England and Wales, a consumer lifestyle survey and the technique of spatial microsimulation (see *Spatial microsimulation*). The 2021 Census counts are provided at the geography of the 36,672 Lower Super Output Areas (LSOA) (Office for National Statistics 2022) each of which typically contains around 1,600 household residents in 700 households. The following variables are used to capture the characteristics of each LSOA:

- Age of Household Reference Person (HRP). The justification is related to the influence that life stage has on pet ownership, capturing the dynamics of the lifestyles of younger populations, the formation of families, and desire for companionship for seniors;
- Occupation of HRP. Capturing differentials associated with wealth or work-life balance opportunities;
- Living Arrangements. The form of household, whether single or living in a couple and the nature of the relationship between the couple (married/co-habiting; divorced or widowed);
- Property type. An important aspect that reflects both wealth/income and availability of outdoor space (i.e. a garden or yard), e.g. detached/semi-detached houses vs terraced housing vs flats/apartments;
- Tenure. Again, an important aspect, with families having greater freedom to own a pet with certain types of tenure (owned/mortgaged) against others (social rented, and especially limited for private rented);
- Number of bedrooms. The final property related aspect is the number of bedrooms in the property, and this captures family size, with households with larger number of bedrooms tending to have larger families.

To reflect possible interaction effects in the relationships between these counts, a number of 2021 Census Ready Made tables are used: Property type by Tenure (RM003), Living arrangements by Age of HRP (RM066), Tenure by Number of bedrooms (RM136), Tenure by Occupation (RM140), and Tenure by Age of HRP (RM201).

The 2021 Census does not collect any information regarding household pet ownership, so instead use is made of a 2021 consumer lifestyle survey provided by the Geographic Data Service (2025) that surveys households to collect the above listed characteristics (Ye & Longley 2024) and also asks if the household owns a pet). These data are, however, unable to provide direct estimates of the pet population within an LSOA since, whilst they are extensive in size, they can only provide a limited number of survey points for each LSOA, and the representativeness of the households providing survey responses to the characteristics of the entire LSOAs households is not guaranteed. The pet population of the LSOA is geo-located at the person population weighted centroid (PWC) of the LSOA.

#### Veterinary care

The number of veterinary surgeons and the location of their practice is used to provide measures of veterinary care capacity. These data are obtained from the Royal College of Veterinary Surgeons and represents the situation in early 2025 (Royal College of Veterinary Surgeons 2025). Locations that serve as solely ‘referral units’ are excluded from these data since they are only available to owners following a referral from another practice. ‘Hybrid’ type establishments that accept both appointments from owners and referrals are included. Starting with 4,759 practices in England and Wales (not including one on the Isles of Scilly), after the removal of referral practices there are 4,679 practices remaining. There are some practices where the number of surgeons is not available (856) and the number of surgeons at these practices are imputed. The imputed value is four, which is the median number of surgeons at practices where this information is available. The location information provided is the full unit postcode of the practice and these are aggregated and geo-located to 4,510 locations within England and Wales.

#### Ease of travel

The journey times between practices and the LSOA PWC are calculated using the osrm package (Giraud 2022) in the R statistical software. This algorithm uses information on free flow road speeds by road type, the presence of one-way streets, and imposes turn and parking penalties on the journey. It does not consider periods of traffic congestion or delays due to traffic signals. To limit the number of calculations, only the car travel times of the 360 straight-line closest LSOAs to each practice are calculated (a potential catchment population of 360 × 1600 = 576,000 people), with the journey time for the remaining LSOAs set to 4 h.

#### Area types

This study will calculate a SPAI for each LSOA in England and Wales. To gain an understanding of how this SPAI varies by the type of neighbourhood, the median SPAI for neighbourhoods of certain types is used. The first area type is a classification of LSOAs based on 2011 Census data, with areas being classified via their socio-demographic and socio-economic make up (Office for National Statistics 2018). The second area type is a measure of multiple deprivation, combining measures of deprivation across several domains (e.g. income, educational attainment and crime) (Abel *et al.* 2016; Ministry of Housing Communities and Local Government 2019). The third is an urban/rural classification (Office for National Statistics 2025).

### Methods

#### Spatial microsimulation

As noted above, the consumer survey, which includes pet ownership, has insufficient coverage and contains too many potential biases to be able to provide a reliable estimate of pet ownership at an LSOA neighbourhood geography. A range of techniques in the area of synthetic population estimation are available to provide such estimates (Chapuis *et al.* 2022). One family of synthesis techniques are spatial microsimulation models that have the ability to link domain-detailed survey data to geographically comprehensive Census data, and effectively provide neighbourhood estimates of missing data (in our case pet ownership rates) (Ballas & Clarke 2009; Tanton 2014). The correspondence is made through matching common ‘constraint’ variables, variables that must be available in both the Census and the survey data-sets (in our case the variables listed *Pet population*) and be related to the required outcome, here household pet ownership. Once this correspondence is established, then the full richness of detail from the survey data is available at the detailed geography of the Census.

The model used here is a combinational optimisation technique which is a kind of ‘cloning’ exercise. Households from the consumer lifestyle survey are selected, with replacement, to populate each LSOA. Then, how well these cloned households represent the known Census counts of the constraint variables is assessed. If the correspondence is acceptable then the method finishes. If the correspondence is not acceptable then a sampled household is replaced with another and a re-assessment made. This continues until either an acceptable solution is found, or a fixed number of replacements have been made. When this technique finishes, any non-Census variables in the cloned households (e.g. household pet ownership) can be used as estimates for the LSOAs. Similar approaches have been used to estimate a wide range of missing data at the small-area level, such as smoking rates (Tomintz *et al.* 2009), mental health/depression (Morrissey *et al.* 2010), obesity (Edwards *et al.* 2011), food, drink and tobacco expenditure (James *et al.* 2019) and rural farm structures and tax regimes (O’Donoghue *et al.* 2012; O’Donoghue 2017). The software used in this paper to derive the small area estimates can be found in Harland (2013).

#### Floating catchment area

A technique that is able to overcome the problems mentioned in *Accessibility to veterinary care* with some of the existing accessibility measures is the Floating Catchment Area (FCA) technique (Luo & Wang 2003) which has had wide applicability, particularly in health contexts (Langford *et al.* 2016). At the heart of this is the SPAI. The SPAI has the properties that it increases as: the competition for care decreases, the supply of care increases, and the travel between the demand and the care decreases. Therefore, higher values of the SPAI denote better accessibility. This technique was recently used by Neal (2023) for measuring access to veterinary care for the household population of four neighbouring counties in Alabama, USA. The analysis used the E2SFCA variant (Luo & Qi 2009) that enhanced the original formulation by using discrete distance decay weights for travel times. In the discussion, Neal (2023) acknowledged that the E2SFCA may tend to overestimate available resources, necessitating the introduction of a third step to control for this overestimation. Here, we adopt such a third step that takes better account of potential competition between supply locations using the Modified Huff Three Step Floating Catchment Area technique (Subal *et al.* 2021) with a variable catchments modification (MHV3SFCA) (Jörg & Haldimann 2023) that ensures that all neighbourhoods have access to an equal number of practices (here denoted as Q).

The three steps in the calculation of the MHV3SFCA SPAI are described here:

Step 1: Calculation of Huff selection probabilities (Huff_ij_). These probabilities measure the probability of an interaction between a veterinary practice and an LSOA neighbourhood.
(1)

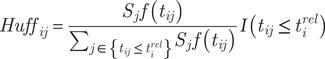

Where i are the demand LSOAs locations;

j are the supply veterinary practice locations;

S_j_ is the service capacity at location j, a count of the number of surgeons in the practice;

f(t_ij_) is the journey time decay weight function (see below);

t_ij_ is the car journey time from i to j;

t_i_^rel^ is the relevant catchment to access the Q closest practices;

I(t_ij_ ≤ t_i_^rel^) is a binary indicator to denote if the journey time is less than t_i_^rel^.

Step 2: Calculation of supply ratios for each practice (R_j_). This is a measure of the available veterinary care that the practice is able to offer, normalised by the catchment population.
(2)

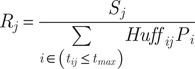

Where P_i_ is the number of pet owning households in LSOA i.

Step 3: Calculation of spatial accessibility index (SPAI_i_). This is the summation of all the available care within the catchment of the LSOA neighbourhood.
(3)

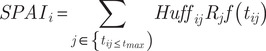

To operationalise this method some assumptions and parameters are required. Here a Gaussian time decay weight function is used:
(4a)

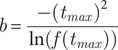

and
(4b)

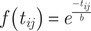

**Figure 1.**
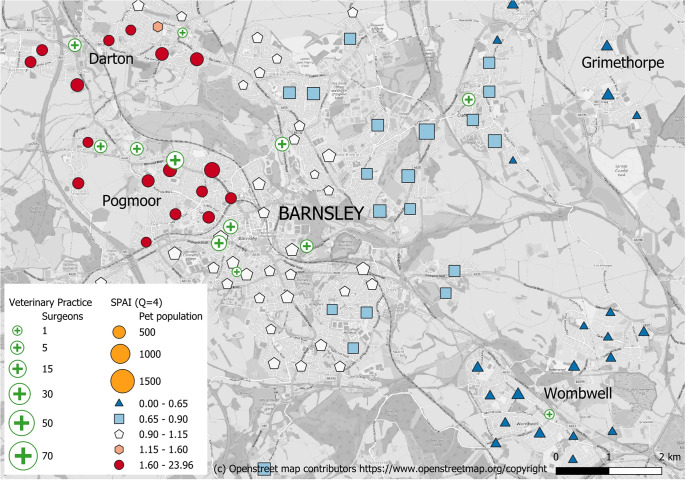
Location of veterinary practices and the Spatial Accessibility Index (SPAI) to the four closest practices (Q) for neighbourhoods in Barnsley, south Yorkshire, England. Higher values of SPAI, shown as red, indicate better accessibility and lower values, shown as blue, indicate poorer accessibility.

To define the shape of this function, two parameters are required, the maximum reasonable travel time (t_max_) and the weight at this time f(t_max_). Here a maximum reasonable travel time of 30 min is used and the weight at this time is 0.01. In a study of veterinary care for food producing animals, Berrada *et al.* (2022) used equivalent travel times of 15; 30; 45 and 60 min and reported that other studies outside the veterinary care sector have settled on a 30-min journey time. In regards to the Q parameter, Langford *et al.* (2021) experimented with various values between 2 and 6, and Jörg and Haldimann, (2023) recommends values up to 10. To help in choosing a suitable value of Q in the context of veterinary care, the CMA (2025b) reports that 51% of respondents considered just one practice, 29% considered two and 15% considered three or more. To allow slightly more choice, a value of Q = 4 (see Figure 1) is used here. The calculations are made using the R code of Hauser (2023).

## Results

The MHV3SFCA is able to calculate a SPAI for all LSOAs in England and Wales. For a national perspective on accessibility to practices, the median SPAIs for various neighbourhood types are provided in Table 1. In general, it can be seen that more affluent and rural locations have better accessibility to veterinary care than deprived and urban locations. To further illustrate the utility of these calculations at a local neighbourhood geography, a case study of a medium-sized town is provided, Barnsley which is located in south Yorkshire, England.

**Table 1.** The number of Neighbourhoods (Lower Super Output Areas - LSOAs) in England and Wales that sit within three typologies: the Area Classification (ONS 2018); quintiles of deprivation; and the six-tier Urban Rural Classification. For each sub type, the number of pet owning households, the percentage of households with pets and Spatial Accessibility Index (SPAI - representing access to veterinary care) are reported. The percentage of pet owning households and SPAI are colour coded where red is a high SPAI and blue is a low SPAI

Source	Number of neighbour-hoods (LSOAs)	Median		
		Number of pet-owning households	Percentage pet households	Accessibility score (SPAI)
**UK Pet Food Association (2021)**			59%	
**Area classification**				
Countryside living	4,519	484	69%	1.47
Industrious communities	7,323	422	62%	1.10
Hard-pressed communities	4,874	411	61%	0.86
Suburban living	6,097	380	60%	1.13
Ethnically diverse professionals	5,387	376	56%	1.05
Cosmopolitan student neighbourhoods	1,212	385	54%	0.89
Multicultural living	3,970	314	47%	0.65
Inner city cosmopolitan	2,289	312	43%	0.81
**Deprivation**				
Most deprived	7,155	390	59%	0.76
More deprived	7,107	393	59%	0.85
Medium deprivation	7,074	404	61%	1.05
Less deprived	7,190	399	60%	1.14
Least deprived	7,145	384	60%	1.27
**Urban/rural (2021)**				
Urban: Nearer to a major town or city	27,106	381	59%	0.91
Urban: Further from a major town or city	2,451	422	62%	1.24
Larger rural: Nearer to a major town or city	2,127	442	63%	1.44
Larger rural: Further from a major town or city	1,021	476	65%	1.56
Smaller rural: Nearer to a major town or city	1,735	456	69%	1.47
Smaller rural: Further from a major town or city	1,231	488	71%	1.43

### Neighbourhood types

For a wider understanding of how the SPAI varies by type of neighbourhood, the median SPAI for various categories of neighbourhood is shown in Table 1.

#### Area classification

Using our estimates, the median count of pet-owning households in each neighbourhood is highest for the two areas classified as Countryside living (with 484 households owing at least one pet), and Industrious communities (422). The lowest numbers are for Multicultural living (314) and Inner-city cosmopolitan (312). The median for the proportion of pet-owning households shows a wide range, highest for Countryside living (69% of households owing at least one pet) and lowest for Inner-city cosmopolitan (43%). For comparison purposes, the UK Pet Food Association (2024) estimates that a proportion of around 59% of households in the UK own at least one pet, which sits comfortably inside this range.

The SPAI values provide a measure of how accessible veterinary care is for these neighbourhoods. If access was equitable, with a balance between the supply of veterinary care and the size of pet populations, there is an expectation that all these values would be roughly similar (this balance is captured by the R_j_ calculation at [2]). This is not the case. It is highest for Countryside living (1.47), showing that neighbourhoods with many pets still have disproportionately good access to veterinary care. For some other neighbourhoods the opposite is the case, for example, Hard-pressed communities have poor accessibility (0.86) for their large pet population (with, on average, 411 households with at least one pet). For all neighbourhood types, accessibility is the poorest for Multicultural living neighbourhoods (0.65).

#### Deprivation

In terms of the median count of pet households and the proportion of pet-owning households by deprivation there is much more consistency here than seen for the Area classification. All neighbourhoods, on average, contain around 400 pet-owning households. However, there are clear differences in the SPAI, with a strong gradient; as deprivation increases, accessibility decreases.

#### Urban/rural

A form of gradient is also apparent on the urban/rural scale. Urban areas have the lowest count of pet-owning households followed by larger rural areas, with smaller rural areas having the highest number of pet households. Depending on the proximity to a major town or city, there are more pet-owning households the further away from the town or city. The SPAI is generally highest for rural areas, in particular the larger rural areas that are further away from a town or city, which has the highest SPAI of any neighbourhood type seen in this table.

### Case study

The town of Barnsley in south Yorkshire, England, has a population of around 244,600 people residing in 108,000 households. Using the spatial microsimulation technique, we estimate that 67,646 households have a pet (i.e. 63% of households). Figure 1 shows the SPAI values for all the neighbourhoods in Barnsley, with hot spots of good accessibility shown in red and cold spots of poorer accessibility in blue. The size of the shapes represents the size of the neighbourhood pet population (number of pet-owning households) whilst the colour and shape gives the value of the SPAI. The location of veterinary practices are also shown, and their size is proportional to the number of veterinary surgeons working at the practice.

The SPAI values are generally average in the town centre, with a balance typically set between the size of the pet population and veterinary care capacity. In contrast, the SPAI is particularly high in the suburbs of Pogmoor to the east, and Darton to the north of the town centre. Two suburbs that do poorly are Wombwell to the south-east and Grimethorpe to the north-west. Wombwell has a sizeable pet population (10,941 households and 6,824 with pets, which is generally high, but typical for Barnsley, 63% of households owning a pet) but only one practice with a single surgeon. Both Wombwell and Grimethorpe are also a considerable travel time away from additional practices. These three factors combined mean that all 15 of Wombwell’s neighbourhoods have SPAIs that are in the lowest 10% nationally.

## Discussion

In this study we have used a spatially detailed estimate of the number of pet-owning households and the number of veterinary surgeons available at practices to calculate a spatial accessibility index for neighbourhoods in England and Wales. We make a unique contribution by providing spatially detailed estimates of the demand and supply for veterinary care, which has strong implications for the welfare of companion animals in different spatial and demographic contexts.

A preliminary task entailed estimating the spatial distribution of pet-owning households, since no existing estimates were available that covered the entire pet population at the detailed local scale required. These estimates are in general accordance with expectations from the literature. Pet ownership was highest in more rural and sub-urban neighbourhoods, and lowest in inner cities. This is likely driven by three factors. First, the type and tenure of housing stock, with inner city housing stock being predominately composed of terraces or flats with limited outdoor space. These properties are also often rented (social or private rental), with pet ownership commonly prohibited by the rental contract. In contrast, rural and suburban neighbourhoods are likely to have access to gardens, and either own their property or are paying for it through a mortgage. Second, if no garden is available to the household, then a nearby park or green space may be required to exercise a dog. In inner city locations land is more valuable and at a premium meaning that parks are less common, or those that are available are overcrowded (Shoari *et al.* 2020) or may have safety concerns for their use (Hobbs *et al.* 2017). Finally, there is the life stage and occupation of the residents in the neighbourhood, with single, younger, urban-dwelling individuals less likely to own a pet such as a dog or cat, whilst families living in the suburbs or working in the countryside more able to be time flexible, and to accommodate a cat or dog as a pet.

The calculation of the SPAIs revealed some interesting findings. The SPAI was highest in the least deprived (1.27) and more rural locations (1.43 to 1.56). Given the high demand for veterinary care in such locations (see the pet estimates summarised in Table 1) one might consider that this high demand would have the potential to adversely affect the SPAI for such locations, via step 2 of the MHV3SFCA calculation. This is not the case, and the available supply has not only been maintained relative to the demand but is actually disproportionately better than for urban neighbourhoods. In particular, the larger rural areas that are a considerable distance from a major town or city have both a large pet population and also good accessibility to veterinary care. In rural locations too, the time taken to travel from the neighbourhood to obtain a service usually penalises such locations in accessibility calculations. However, this extra travel time appears to have been compensated for with enhanced quantity and location of veterinary care (Department of Environment, Food and Rural Affairs 2022). In their study of the current position and ambitions for recent graduates, Kinnison and May (2013) found correlations between their sex and childhood upbringing, and where they chose to practice. Graduates from rural neighbourhoods were more likely to want to practice in rural locations – and 70% of the surveyed graduates were from small towns or rural neighbourhoods. This preference may indicate a differential in the supply of veterinary care in such areas. The accessibility in more deprived neighbourhoods is seen to be poor, with similar levels of pet ownership to other neighbourhoods but with fewer veterinary practices being available, and this despite their generally better journey time options. These estimates show that such deprived neighbourhoods are likely to have less choice of practice, and therefore capacity and competition in veterinary care will be poorer.

### Policy

Handy and Niemeier (1997) recommend that to aid interpretability, rather than focusing on absolute levels of accessibility it is more instructive to focus upon relative levels. This leads us to advocate that a veterinary desert can be reasonably identified as an LSOA with a SPAI in the bottom 1% of all LSOAs. In such LSOAs the competition between practices would be low and pet-owning households in these locations may be unable to ‘shop around’ for alternatives.

For a wider perspective regarding veterinary deserts, we can return to the definition provided by Bunke *et al.* (2024) whereby a veterinary desert should include three themes: accessibility; affordability; and availability. The SPAI explicitly captures two of these three themes, accessibility through locations and journey times and availability through the number of veterinary surgeons. What is not explicitly accounted for is the affordability. However, in Table 1 we show that it is possible to relate the SPAI to levels of deprivation. This means that a composite definition of a veterinary desert can be made by jointly considering both the quintile of SPAI for an LSOA and its quintile of deprivation. This shares similarities with findings elsewhere, for example, Wang and Ramroop (2018) who used a joint accessibility-vulnerability measure to examine accessibility to pharmacies.

Once the location of veterinary deserts has been identified then the impact on how accessibility may change through the provision of extra care capacity can be measured using the MHV3SFCA approach. This can be captured by employing more surgeons at existing practices or the establishment of new practices. Also, it is possible to examine sub-markets, for example, the PDSA organisation provides free or heavily subsidised care through its network of 48 pet hospitals and by restricting the veterinary practices to only these hospitals, accessibility to this kind of care for a sub-population can be evaluated by using MHV3SFCA.

Whilst this study has been limited to England and Wales there is nothing intrinsic to the application of the MHV3SFCA that dictated this choice. All that is required are suitable measures of demand, supply and impedances (journey times) that connect the demand and supply locations. This means that this study can be easily replicated to other countries and at different geographic scales.

### Study limitations

Geographic accessibility is merely one barrier to obtaining pet care. As Bunke *et al.* (2024) identified in their review study, cost is important, both from the perspective of owners’ income and the cost of care. Another aspect is the level of the owner’s education and how this correlates with health literacy in both the human and pet domains. Croney *et al.* (2025) also provide commentary on the interactions between economic and sociocultural barriers to care, and animal welfare. In the UK, the CMA (2025a) lists some experiences of pet owners that have the potential to impact how and where owners seek out care, for example: the lack of compassion/understanding/care; being dissatisfied with the veterinarian; lack of convenient appointments; and change of practice ownership. These broader barriers are not explicitly accounted for in the SPAI calculations provide here.

One data limitation is our inability to establish a contemporary time-frame for our pet population estimates, which are based on 2021 Census and consumer lifestyle data, and the supply of veterinary care from 2025. It is, however, reasonable to assume that there is a ‘temporal stability’ in pet ownership, with households retaining pets, particularly dogs and cats, for many years, or through the replacement of deceased pets. To quantify the extent of this behaviour, the CMA (2025a) study reports that 24% of respondents have been pet owners for 4–10 years, another 23% for 11–20 years and 27% for over 30 years. Thus, 2024 or 2025 estimates of pet-owning households would be similar to those here for 2021. If consumer lifestyle data containing information on household pet ownership become available after 2021, updated pet household estimates would be possible.

Here, the capacity for care is measured using the number of veterinary surgeons in the practice. Information is also available on the number of veterinary nurses in the practice. With such nurses becoming increasingly skilled (Vivian *et al.* 2022), a refinement to this analysis could be to incorporate this capacity into the calculation. This could be by computing a weighted sum of care capacity, although what the relative weights should be between a surgeon member of staff and a nurse is a matter for debate.

**Figure 2.**
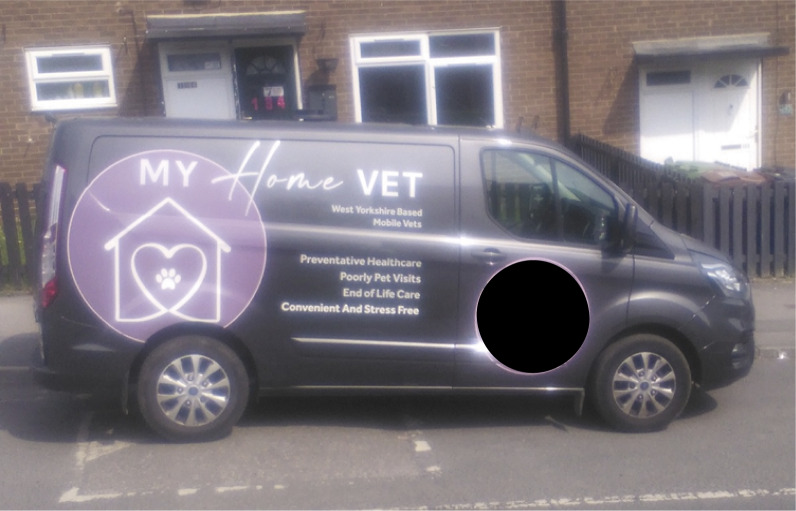
A mobile veterinary van in the UK.

There are variations regarding the kind of assessment that this study has undertaken (LaVallee *et al.* 2017). On the demand size, there are certain pet-owning populations that have not been explicitly considered in this study, particularly homeless people (Williams & Hogg 2016). On the supply side, there are telemedicine (Teller & Moberly 2020) and mobile veterinarian (see Figure 2) options. These two supply side aspects are part of the range of potential solutions to address gaps in access to veterinary care covered by Croney *et al.* (2025). Since these services will supplement traditional brick-and-mortar practices it is likely that their availability will improve accessibility for locations where these options are available.

### Animal welfare implications

Pet owners have a responsibility to provide care for their animals. One important aspect of this is veterinary care. Failure to access this care may have significant adverse impacts on the pet’s physical and/or behavioural welfare status (Mellor 2016) and unless this care is provided in a timely fashion, these issues may escalate (Croney *et al.* 2025). Additionally, where pets socialise with other pets, there is also the risk that this lack of care increases the burden of disease in the wider pet population.

Whilst there are many aspects that may affect the level of care provided, ranging from the specifics of the practice environment (Dawson *et al.* 2016) to more societal issues (Muldoon & Williams 2024c), two significant aspects are the cost and accessibility of care. These two factors can be related, with the lack of available local options for pet healthcare impeding market competition and thereby increasing costs. This study succeeds in computing a theoretically robust estimate of how the accessibility of veterinary practices varies by neighbourhood, taking account of the number of veterinarians, the potential for competition for services and the ease of travel. Using a relative measure of accessibility to identify those (1%) of LSOAs with the lowest values enables locations where veterinary deserts may exist to be identified. With this knowledge, extra provision may be deployed in such neighbourhoods in order to optimally enhance accessibility and thereby promote competition, and provide more choice at, hopefully, a lower cost.
